# Impact of a Structured MRI Reporting Template on the Completeness of Primary Rectal Cancer Staging Reports: A Retrospective Audit

**DOI:** 10.7759/cureus.112190

**Published:** 2026-07-06

**Authors:** Manish Kumar Jha, Shilpi Rani, Kashi Nath Sarkar, Shivani Sarkar, Manisha Sarkar

**Affiliations:** 1 Department of Radiodiagnosis, JIS School of Medical Science & Research, Howrah, IND; 2 Department of Anatomy, Rama Medical College Hospital & Research Centre, Hapur, IND; 3 Department of Radiodiagnosis, Balco Medical Centre, Vedanta Medical Research Foundation, Naya Raipur, IND; 4 Department of Radiodiagnosis, Raipur Institute of Medical Sciences, Godhi, IND; 5 Department of Oncopathology, Tata Medical Center, Kolkata, IND; 6 Department of Community Medicine, Bankura Sammilani Medical College and Hospital, Bankura, IND

**Keywords:** circumferential resection margin (crm), extramural venous invasion, mri staging, quality improvement research, rectal cancer, rectal mri, report completeness, structured reporting template

## Abstract

Introduction: High-resolution pelvic magnetic resonance imaging (MRI) is essential for local staging of primary rectal carcinoma and for communicating surgically relevant risk factors before multidisciplinary treatment planning. Free-text MRI reports may describe the primary tumor but may inconsistently document key management-relevant findings, including mesorectal fascia and circumferential resection margin status, depth of extramural spread, extramural vascular invasion, lateral pelvic lymph nodes, and low rectal sphincter complex involvement. This retrospective audit assessed whether use of a structured MRI reporting template was associated with improved completeness of primary rectal cancer staging reports.

Methods: This retrospective audit included 60 pelvic MRI reports performed for suspected or biopsy-proven primary rectal carcinoma at a tertiary care radiology department. Thirty consecutive reports prepared before template implementation were assigned to the free-text reporting group, and 30 reports prepared after template implementation were assigned to the structured reporting group. Reports were assessed using a predefined checklist of essential MRI staging elements, including tumor location, distance from the anal verge, craniocaudal tumor length, circumferential tumor position, relationship to the anorectal junction and anterior peritoneal reflection, MRI T category, depth of extramural spread, mesorectal fascia/circumferential resection margin status, extramural vascular invasion, mesorectal nodes, tumor deposits, lateral pelvic lymph nodes, sphincter complex involvement, levator ani involvement, adjacent organ invasion, and final MRI-based risk summary. The primary and only measured outcome was report completeness.

Results: The mean completeness score was higher in the structured reporting group than in the free-text group, with mean scores of 91.3% and 58.6%, respectively. Structured reports showed more complete documentation of several clinically important staging domains that were inconsistently recorded in free-text reports, including mesorectal fascia/circumferential resection margin relationship, depth of extramural spread, extramural vascular invasion, lateral pelvic nodal assessment, and low rectal sphincter complex assessment. Structured reports also more frequently included a concise MRI-based risk summary integrating tumor level, T stage, nodal status, margin risk, extramural vascular invasion, and anticipated surgical relevance. No formal statistical hypothesis testing was performed; therefore, these findings should be interpreted descriptively rather than inferentially.

Conclusions: Implementation of a structured MRI reporting template was associated with higher report completeness in this descriptive retrospective audit of primary rectal cancer staging reports. The structured template was associated with more complete documentation of clinically relevant reporting elements, particularly margin status, extramural spread, extramural vascular invasion, lateral pelvic nodes, and low rectal sphincter complex involvement. However, report completeness was the only measured outcome, and no formal statistical testing was performed. Further studies with statistical testing, reviewer blinding, and assessment of diagnostic accuracy or clinical outcomes are needed.

## Introduction

High-resolution pelvic magnetic resonance imaging (MRI) is a key investigation in the initial staging of primary rectal carcinoma. Its value lies not only in assigning a local T and N category, but also in defining the anatomical details that influence multidisciplinary decision-making. A clinically useful rectal MRI report should communicate tumor height, craniocaudal length, circumferential location, relationship to the anorectal junction and anterior peritoneal reflection, depth of extramural spread, relationship to the mesorectal fascia, predicted circumferential resection margin (CRM) status, extramural vascular invasion (EMVI), mesorectal and lateral pelvic nodal disease, sphincter complex involvement, levator ani involvement, and adjacent organ or pelvic sidewall invasion [1-4]. These findings help colorectal surgeons, radiation oncologists, and medical oncologists determine the risk category of the disease and plan appropriate treatment.

The importance of MRI in rectal cancer staging is closely related to its ability to identify surgically relevant risk factors before treatment. Assessment of the mesorectal fascia and predicted CRM is one of the most important components of the report, as a threatened or involved margin can influence the need for neoadjuvant treatment and the expected surgical plane [5,6]. Similarly, MRI-detected EMVI is an adverse imaging feature and should be stated clearly as present, absent, or equivocal rather than left unmentioned [7]. In low rectal tumors, reporting should extend beyond conventional T staging and should specifically describe the internal sphincter, intersphincteric plane, external sphincter, puborectalis, levator ani, and ischioanal fossa when relevant, because these findings affect the feasibility of sphincter-preserving surgery and the choice of operative approach [8].

Despite the availability of consensus recommendations and synoptic reporting guidance, free-text MRI reports may vary considerably in structure and content. In routine practice, a free-text report may describe the rectal mass and broad stage, but may omit important negative findings such as absence of EMVI, clear CRM, non-involvement of the sphincter complex, or absence of suspicious lateral pelvic nodes. Such omissions do not always mean that the structures were abnormal or unassessed; however, they can reduce the clarity of the report during tumor board discussion and may require additional clarification from the reporting radiologist. This is particularly relevant in institutions where rectal MRI examinations are reported by radiologists with variable exposure to gastrointestinal oncology imaging.

Structured reporting has been proposed as a practical method to improve consistency and completeness in rectal cancer MRI. A structured template prompts the radiologist to evaluate each relevant anatomical compartment in sequence and to document both positive and clinically important negative findings. It also encourages uniform terminology for CRM status, EMVI, nodal compartments, tumor deposits, and low rectal sphincter involvement. Importantly, structured reporting does not replace image interpretation or radiologist judgment; rather, it provides a framework that reduces the chance of omitting management-relevant information.

The present study was conducted as a retrospective descriptive audit to evaluate whether implementation of a structured MRI reporting template was associated with higher completeness of primary rectal cancer staging reports. The primary objective was to compare overall report completeness between free-text reports and structured-template reports. The secondary objective was to describe differences in documentation of selected reporting elements, including CRM status, depth of extramural spread, EMVI, lateral pelvic nodal assessment, and low rectal sphincter complex evaluation.

## Materials and methods

This retrospective audit was conducted in the Department of Radiodiagnosis at the Raipur Institute of Medical Sciences, Chhattisgarh, India. The study was approved by the Institutional Ethics Committee, Raipur Institute of Medical Sciences (approval number: IEC/RIMS/2025/42). The study was based on de-identified radiology report-level data and did not involve direct patient contact, additional imaging, change in treatment, or any prospective intervention. Individual informed consent was waived by the Institutional Ethics Committee because this was a retrospective audit based on de-identified radiology report-level data, with no direct patient contact, additional imaging, or change in treatment.

Study process

**Figure 1:**
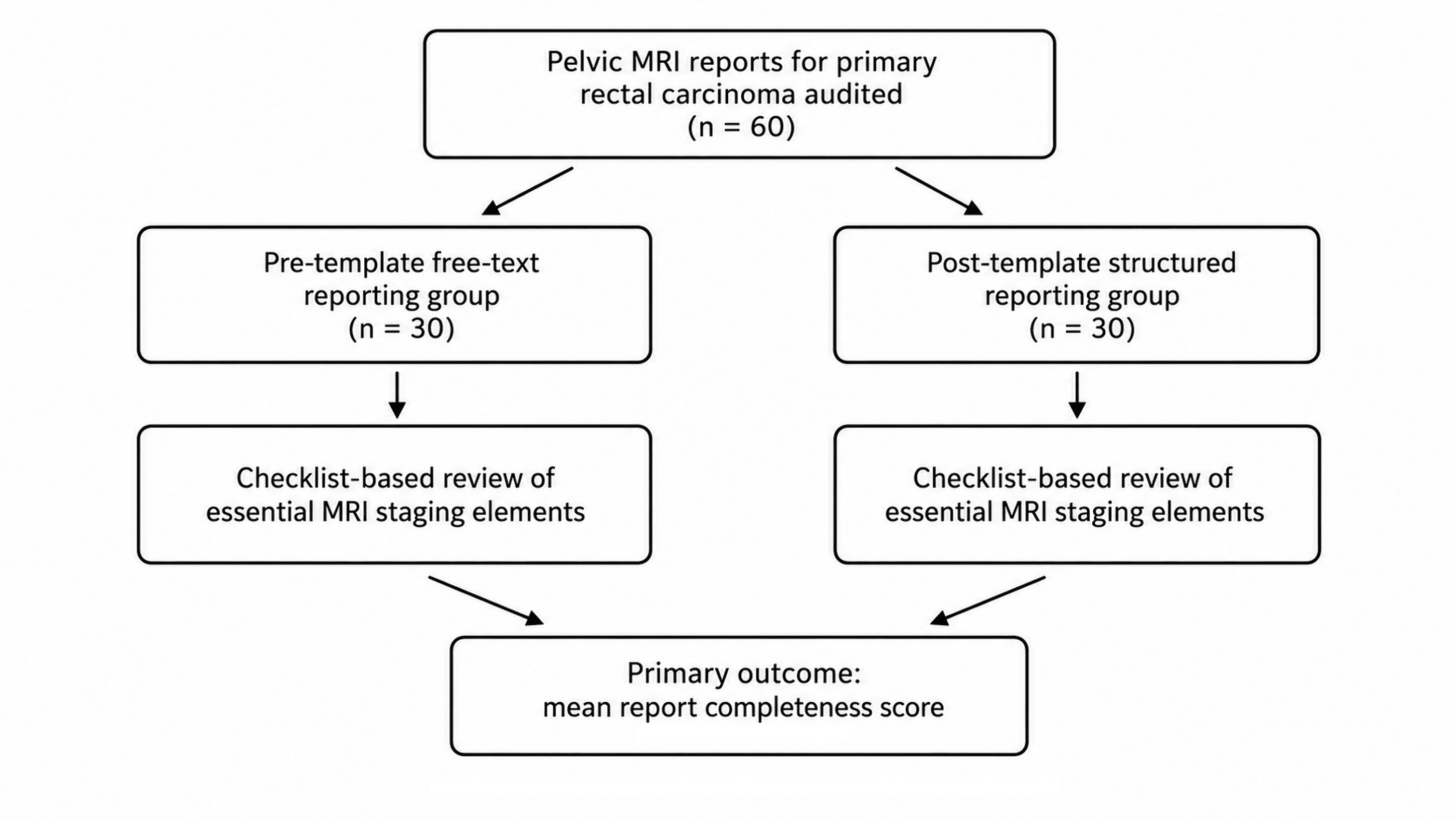
Audit design and report allocation Flow diagram showing the retrospective audit design. Sixty pelvic MRI reports performed for primary rectal carcinoma were divided into two groups: 30 pre-template free-text reports and 30 post-template structured-template reports. Each report was assessed using a predefined checklist of essential MRI staging elements, and the mean completeness score was compared between the two groups. MRI, magnetic resonance imaging

The audit included 60 pelvic MRI reports performed for suspected or biopsy-proven primary rectal carcinoma, with 30 reports in the pre-template free-text group and 30 reports in the post-template structured reporting group. The pre-template group included 30 consecutive MRI reports prepared in a conventional free-text format before implementation of a structured rectal MRI reporting template. The post-template group included 30 consecutive MRI reports prepared after implementation of the structured template. The structured MRI reporting template was implemented on January 1, 2026. The pre-template period was from July 1, 2025, to December 31, 2025, and the post-template period was from January 1, 2026, to June 19, 2026. The overall audit design and group allocation are summarized in Figure 1. 

Eligibility

Reports were included when the MRI examination was performed for primary local staging of rectal carcinoma. Reports were excluded if the MRI was performed for post-neoadjuvant response assessment, postoperative surveillance, suspected recurrence, non-rectal pelvic malignancy, incomplete examination, unavailable report, or inadequate documentation preventing assessment of reporting completeness.

MRI procedure and assessment

MRI examinations were performed using a dedicated pelvic MRI protocol. The standard protocol included sagittal T2-weighted imaging for tumor localization and planning of tumor-oriented planes, high-resolution oblique axial T2-weighted imaging perpendicular to the tumor axis, oblique coronal T2-weighted imaging when required for low rectal tumors, axial diffusion-weighted imaging (DWI), and apparent diffusion coefficient maps. Intravenous contrast was not considered mandatory for inclusion in the audit and was used selectively according to the clinical indication and departmental protocol. The audit focused on the content of the final radiology report rather than on independent reinterpretation of imaging findings.

A predefined checklist of essential primary rectal cancer MRI reporting elements was used for assessment. The checklist included tumor location, distance from the anal verge, craniocaudal tumor length, circumferential tumor position, relationship to the anorectal junction, relationship to the anterior peritoneal reflection, MRI T category, depth of extramural spread, mesorectal fascia and predicted circumferential resection margin status, extramural vascular invasion, mesorectal nodal status, tumor deposits, lateral pelvic lymph node assessment, sphincter complex involvement, levator ani involvement, adjacent organ or pelvic sidewall invasion, and final MRI-based risk summary. The reporting checklist used for completeness assessment is shown in Table 1. These elements were selected because they are routinely required for multidisciplinary rectal cancer staging and preoperative planning [1-4].

**Table 1:** Checklist of essential MRI reporting elements for primary rectal cancer staging Checklist used to assess completeness of free-text and structured-template MRI reports. Each item was scored as documented when it was explicitly mentioned as present, absent, or equivocal. Case-specific elements were assessed only when applicable to tumor location. CRM, circumferential resection margin; EMVI, extramural vascular invasion; MRI, magnetic resonance imaging

Reporting Element	Adequate Documentation	Applicability
Tumor location	Low, mid, or upper rectal location documented	All cases
Distance from anal verge	Lower tumor margin distance stated in centimeters	All assessable cases
Craniocaudal tumor length	Longitudinal tumor length documented	All cases
Circumferential tumor position	Clock-face position or involved rectal wall described	All cases
Anorectal junction relationship	Relationship to the anorectal junction stated	All cases
Anterior peritoneal reflection	Tumor described in relation to the anterior peritoneal reflection	Mid and upper rectal tumors
MRI T category	MRI-based local T category documented	All cases
Depth of extramural spread	Depth of extramural extension stated in millimeters	T3 tumors
Mesorectal fascia and CRM status	Margin described as clear, threatened, or involved	All cases
Extramural vascular invasion	EMVI stated as present, absent, or equivocal	All cases
Mesorectal nodes	Suspicious mesorectal nodes documented or absence stated	All cases
Tumor deposits	Tumor deposits documented as present or absent	All cases
Lateral pelvic nodes	Internal iliac, obturator, or lateral pelvic nodal status documented	All cases
Sphincter complex involvement	Internal sphincter, intersphincteric plane, external sphincter, or puborectalis assessed	Low rectal tumors
Levator ani involvement	Levator ani involvement stated as present or absent	Low rectal tumors
Adjacent organ or pelvic sidewall invasion	Invasion stated as absent, equivocal, or definite	All cases
Final MRI-based risk summary	Concise staging and risk summary included in impression	All cases

Each report was reviewed against the checklist. A reporting element was scored as present when it was explicitly documented in the report, either as a positive finding, a negative finding, or a clearly stated equivocal finding. An element was scored as absent when it was not mentioned. For case-specific elements, such as sphincter complex and levator ani assessment in low rectal tumors, the item was assessed when applicable. The completeness score for each report was calculated as the number of documented applicable elements divided by the total number of applicable elements, expressed as a percentage. The primary outcome measure was the mean report completeness score in the free-text and structured-template groups.

The secondary outcome measures were documentation rates of individual MRI staging elements, with particular attention to mesorectal fascia/circumferential resection margin status, depth of extramural spread, extramural vascular invasion, lateral pelvic lymph nodes, low rectal sphincter complex assessment, levator ani assessment, and final MRI-based risk summary. These elements were emphasized because omission of these details may affect tumor board interpretation and surgical or oncologic planning. 

Statistical analysis

Data were entered into a spreadsheet (Microsoft Corporation, Redmond, Washington, United States) and summarized using descriptive statistics. Continuous variables were expressed as mean values where appropriate, and categorical variables were expressed as frequencies and percentages. The mean completeness score before and after structured template implementation was compared descriptively between the two groups. No formal statistical hypothesis testing was performed. The audit was designed as a descriptive quality-improvement assessment, and report completeness was the only measured outcome. Therefore, the findings should be interpreted descriptively rather than inferentially, and not as evidence of statistical significance or clinical outcome difference.

## Results

A total of 60 pelvic MRI reports performed for primary rectal cancer staging were included in the audit. Thirty reports were evaluated in the pre-template free-text group, and 30 reports were evaluated in the post-template structured reporting group. All included reports were assessed using the predefined reporting checklist shown in Table 1.

The mean report completeness score was higher in the structured-template group than in the free-text group, with mean scores of 91.3% and 58.6%, respectively. This represented an absolute descriptive difference of 32.7 percentage points after structured template implementation. As no formal statistical testing was performed, this difference should be interpreted as descriptive rather than inferential. Structured reports showed more consistent documentation of both positive findings and clinically important negative findings, particularly in domains relevant to surgical planning and multidisciplinary discussion.

Higher documentation rates were observed for mesorectal fascia and circumferential resection margin status, depth of extramural spread, extramural vascular invasion, lateral pelvic lymph node assessment, and low rectal sphincter complex assessment. In the free-text group, these findings were variably described and were sometimes omitted when negative. In the structured-template group, these elements were more consistently documented as present, absent, or equivocal, which improved the interpretability of reports during staging review. The comparison of individual reporting elements between the free-text and structured-template groups is summarized in Table 2.

**Table 2:** Comparison of report completeness before and after structured template implementation Comparison of documentation rates for essential primary rectal cancer MRI reporting domains in free-text and structured-template reports. Values are shown as the number of reports with explicit documentation and percentage, unless otherwise stated. CRM, circumferential resection margin; MRI, magnetic resonance imaging.

Reporting Domain	Free-text Reports (n = 30)	Structured-template Reports (n = 30)	Absolute Improvement
Overall mean completeness score	58.6%	91.3%	32.7 percentage points
Tumor location documented	26 (86.7%)	30 (100.0%)	13.3 percentage points
Distance from anal verge documented	20 (66.7%)	29 (96.7%)	30.0 percentage points
Craniocaudal tumor length documented	18 (60.0%)	28 (93.3%)	33.3 percentage points
Circumferential tumor position documented	12 (40.0%)	27 (90.0%)	50.0 percentage points
Anorectal junction relationship documented	14 (46.7%)	27 (90.0%)	43.3 percentage points
Anterior peritoneal reflection relationship documented	10 (33.3%)	25 (83.3%)	50.0 percentage points
MRI T category documented	27 (90.0%)	30 (100.0%)	10.0 percentage points
Depth of extramural spread documented for T3 disease	11 (36.7%)	26 (86.7%)	50.0 percentage points
Mesorectal fascia and CRM status documented	15 (50.0%)	29 (96.7%)	46.7 percentage points
Extramural vascular invasion documented	12 (40.0%)	28 (93.3%)	53.3 percentage points
Mesorectal nodal status documented	24 (80.0%)	30 (100.0%)	20.0 percentage points
Tumor deposits documented	8 (26.7%)	25 (83.3%)	56.6 percentage points
Lateral pelvic nodal assessment documented	9 (30.0%)	27 (90.0%)	60.0 percentage points
Sphincter complex assessment documented where applicable	10 (33.3%)	24 (80.0%)	46.7 percentage points
Levator ani involvement documented where applicable	9 (30.0%)	24 (80.0%)	50.0 percentage points
Adjacent organ or pelvic sidewall invasion documented	17 (56.7%)	28 (93.3%)	36.6 percentage points
Final MRI-based risk summary included	7 (23.3%)	27 (90.0%)	66.7 percentage points

The inclusion of a final MRI-based risk summary also improved after the implementation of the structured template. Structured reports more frequently provided a concise impression integrating tumor level, MRI T category, nodal status, mesorectal fascia or circumferential resection margin relationship, extramural vascular invasion, lateral pelvic nodal status, and low rectal sphincter or levator involvement when applicable. This was particularly useful in mid and low rectal tumors, where incomplete description of margin risk, nodal compartments, or sphincter complex anatomy may limit the clinical value of the report.

Overall, structured reporting was associated with higher completeness and more consistent documentation of primary rectal cancer MRI reporting elements in this descriptive audit.

## Discussion

This retrospective descriptive audit found that structured-template reports had higher completeness scores than free-text reports. The mean completeness score was 58.6% in free-text reports and 91.3% in structured-template reports. Higher documentation rates were most apparent in staging domains that are clinically important but commonly under-reported in routine free-text practice, including mesorectal fascia and circumferential resection margin assessment, depth of extramural spread, extramural vascular invasion, lateral pelvic nodal assessment, and low rectal sphincter complex evaluation.

The findings are clinically relevant because rectal MRI reports are used directly in multidisciplinary decision-making. For primary rectal carcinoma, the report should not be limited to a broad T and N category. It should describe the tumor level, relationship to the anorectal junction and anterior peritoneal reflection, depth of extramural spread, predicted circumferential resection margin status, extramural vascular invasion, suspicious nodal compartments, tumor deposits, sphincter complex involvement, levator ani involvement, and adjacent organ or pelvic sidewall invasion [1-4]. These findings influence risk stratification, neoadjuvant treatment planning, radiotherapy fields, and the surgical approach.

One of the important benefits of structured reporting in this audit was more consistent documentation of the mesorectal fascia and predicted circumferential resection margin. MRI assessment of the circumferential resection margin is one of the most important contributions of preoperative rectal imaging, as a threatened or involved margin may alter treatment strategy and surgical planning [5,6]. In free-text reports, the mesorectal fascia may be mentioned only when clearly involved, whereas a structured template encourages the radiologist to document whether the margin is clear, threatened, or involved. This is useful because a clearly documented negative margin is also clinically valuable.

Structured reporting also improved documentation of extramural vascular invasion. MRI-detected extramural vascular invasion is an adverse imaging feature and should be reported as present, absent, or equivocal [7]. In conventional free-text reporting, EMVI may be omitted when not suspected. However, omission can be ambiguous because the clinician cannot determine whether the finding was assessed and absent or simply not addressed. A structured template reduces this ambiguity and provides a more complete staging summary.

Low rectal cancer reporting also benefited from template-based assessment. In low tumors, conventional T staging alone does not provide enough information for operative planning. The report should describe the internal sphincter, intersphincteric plane, external sphincter, puborectalis, levator ani, and ischioanal fossa when relevant [8]. These details help determine the feasibility of sphincter-preserving surgery and whether a more extensive operative approach may be needed. In the present audit, structured reports more consistently included sphincter complex and levator ani assessment than free-text reports.

The descriptive difference observed in this audit suggests that structured reporting may be useful as a quality-improvement tool, although this finding should not be interpreted as inferential evidence because no formal statistical testing was performed. A template does not replace the radiologist’s interpretation, and it does not guarantee diagnostic accuracy by itself. Its role is to ensure that important staging domains are actively considered and explicitly documented. This is particularly useful in departments where rectal MRI examinations are reported by radiologists with variable subspecialty exposure or where reports are reviewed in multidisciplinary tumor boards.

Several potential confounding factors should be considered when interpreting these findings. The higher completeness score observed in the structured-template group may not be attributable solely to the reporting template. It may also reflect increased radiologist awareness after template implementation, temporal changes in departmental reporting practice, variation in radiologist experience or subspecialty exposure, possible differences in case complexity between the pre-template and post-template groups, and a possible Hawthorne effect. Because this was a retrospective non-randomized audit, these potential confounders could not be fully controlled.

A practical structured reporting template used for primary rectal cancer MRI staging is provided in Table 3. This template may be adapted according to local practice, scanner protocol, and multidisciplinary requirements. The most important principle is that the final report should provide a clear preoperative roadmap, including tumor level, T category, margin status, EMVI, nodal compartments, low rectal sphincter anatomy when applicable, and a concise MRI-based risk summary.

**Table 3:** Structured MRI reporting template for primary rectal cancer staging The template is intended to improve the completeness and consistency of staging reports and may be adapted according to institutional protocol and multidisciplinary requirements. CRM, circumferential resection margin; EMVI, extramural vascular invasion; MRI, magnetic resonance imaging; MRF, mesorectal fascia.

Section	Reporting Domain	Structured Reporting Item / Suggested Entry	Response Format / Options	Clinical Relevance
Patient/Study	Clinical indication	MRI pelvis for primary staging of suspected or biopsy-proven rectal carcinoma	Primary staging / preoperative staging	Defines the report as baseline staging rather than restaging or recurrence assessment
	Prior treatment status	No prior neoadjuvant therapy before this staging MRI	Yes / No / Not known	Avoids confusing primary staging with post-treatment response assessment
Technique	MRI protocol	Sagittal T2, high-resolution oblique axial T2 perpendicular to tumor axis, oblique coronal T2 when low tumor, DWI and ADC maps	Protocol completed / limited study	Confirms adequate sequences for local staging
	Study limitation	Motion, incomplete distension, poor plane selection, artifact, or no significant limitation	None / mild / moderate / severe	Documents confidence and need for repeat or problem-solving imaging
Tumor Localization	Tumor location	Lower, mid, or upper rectum	Low / mid / upper	Essential for treatment planning and surgical approach
	Distance from anal verge	Distance from anal verge to lower tumor margin	Centimeters	Determines tumor level and need for low rectal cancer assessment
	Distance from anorectal junction	Tumor above, at, or below anorectal junction	Above / at / below	Helps define low rectal tumor anatomy
	Craniocaudal tumor length	Maximum longitudinal length of tumor	Centimeters	Supports staging description and radiation planning
	Circumferential position	Clock-face location or involved rectal wall	Clock-face / anterior / posterior / lateral / circumferential	Communicates tumor orientation and relation to threatened margin
	Anterior peritoneal reflection	Tumor below, straddling, or above anterior peritoneal reflection	Below / straddling / above / not assessable	Important for peritonealized versus extraperitoneal rectum and surgical planning
Tumor Morphology	Tumor morphology	Annular, semi-annular, polypoidal, ulceroinfiltrative, mucinous features, or stenosing morphology	Descriptive text	Provides morphological context and alerts to mucinous features
Local T Stage	MRI T category	MRI local T category based on depth of invasion and involved structures	T1/T2 / T3 / T4a / T4b	Core MRI staging element
	Depth of extramural spread	Maximum extramural extension beyond muscularis propria	Millimeters; applicable to T3 tumors	Risk stratification and treatment planning
	T3 substage	Substage based on extramural spread depth where used locally	T3a / T3b / T3c / T3d	Adds granularity to T3 disease risk
	T4a criterion	Involvement of visceral peritoneal reflection when present	Present / absent / equivocal	Identifies peritoneal involvement
	T4b criterion	Direct invasion of adjacent organ or pelvic sidewall	Present / absent / equivocal	Defines locally advanced unresectability or need for extended surgery
Margin Assessment	Mesorectal fascia distance	Shortest distance from tumor, involved node, tumor deposit, or EMVI to mesorectal fascia	Millimeters and source of closest point	Predicts circumferential resection margin risk
	CRM status	Predicted circumferential resection margin categorized from MRI	Clear / threatened / involved	Key preoperative risk feature
	Source of margin threat	Primary tumor, suspicious node, tumor deposit, EMVI, or adjacent organ invasion causing closest margin	Descriptive text	Guides tumor board discussion and radiation planning
EMVI	Extramural vascular invasion	Serpiginous or nodular tumor signal within extramural vessels	Present / absent / equivocal	Adverse prognostic imaging feature
Nodes	Mesorectal nodes	Suspicious mesorectal lymph nodes including size, morphology, and location	Present / absent; largest short axis in mm	Supports N staging and risk stratification
	MRI N category	MRI nodal category based on suspicious regional nodes	N0 / N1 / N2	Core staging element for multidisciplinary planning
	Tumor deposits	Discrete extranodal tumor deposits in mesorectum	Present / absent / equivocal	May threaten margin and increase risk category
	Lateral pelvic nodes	Internal iliac, obturator, external iliac, common iliac, or other lateral compartment nodes	Present / absent; side and station	May influence radiation field or surgical nodal planning
Low Rectal Tumor Assessment	Internal sphincter involvement	Involvement of internal anal sphincter by tumor	Present / absent / not applicable	Important for sphincter preservation
	Intersphincteric plane	Tumor extension into intersphincteric plane	Clear / involved / threatened / not applicable	Defines operative plane in low rectal cancer
	External sphincter involvement	Tumor invasion of external anal sphincter	Present / absent / not applicable	Affects feasibility of sphincter-sparing surgery
	Puborectalis involvement	Tumor involvement of puborectalis sling	Present / absent / not applicable	Relevant to low rectal surgical approach
	Levator ani involvement	Tumor involvement of levator ani muscle	Present / absent / not applicable	May suggest need for extended or extralevator approach
	Ischioanal fossa involvement	Tumor extension into ischioanal or ischiorectal fossa	Present / absent / not applicable	Indicates more extensive local spread
Adjacent Structures	Anterior organ involvement	Prostate, seminal vesicle, vagina, cervix, uterus, bladder, ureter, or pelvic sidewall assessed when relevant	Absent / equivocal / definite; specify organ	Guides extended surgical planning
	Bone or sacral involvement	Sacral, coccygeal, or pelvic bone invasion when suspected	Present / absent / equivocal	Identifies advanced local disease
Additional Findings	Peritoneal disease or ascites	Peritoneal nodules, free fluid, or pelvic deposits	Present / absent	May alter staging and treatment pathway
	Other relevant pelvic finding	Hydroureter, fistula, abscess, synchronous lesion, or incidental actionable finding	Descriptive text	Captures findings relevant to care
Impression	Final MRI-based risk summary	Tumor level, MRI T category, N category, CRM/MRF status, EMVI status, lateral nodes, sphincter/levator status, and adjacent organ invasion	Concise structured impression	Provides clear preoperative roadmap for tumor board
	Suggested report conclusion	Example: MRI stage: mid rectal carcinoma, mrT3b N1, CRM clear, EMVI absent, no suspicious lateral pelvic nodes, sphincter complex not involved	Free text using standard terms	Ensures actionable final summary

This study has a few limitations. First, it was a single-center retrospective audit with a limited sample size. Second, report completeness was the only measured outcome; direct patient outcomes, surgical margin status, recurrence, survival, diagnostic accuracy, and pathological concordance were not evaluated. Third, no formal statistical hypothesis testing was performed, so the findings should be interpreted descriptively rather than inferentially. Fourth, the audit assessed whether report elements were documented, not whether each documented imaging interpretation was pathologically correct. Fifth, reviewer blinding, interobserver agreement, and formal adjudication of disagreements were not assessed. Finally, radiologist-level characteristics, including years of experience and subspecialty exposure, were not analyzed.

Despite these limitations, the descriptive findings suggest that structured reporting may help improve the completeness and consistency of primary rectal cancer MRI reports in routine clinical practice. However, further studies using formal statistical testing, blinded review, interobserver agreement assessment, and evaluation of diagnostic accuracy or clinical outcomes are needed before drawing stronger conclusions.

## Conclusions

Structured MRI reporting was associated with higher report completeness in this descriptive retrospective audit of primary rectal cancer staging reports. After implementation of the template, the mean report completeness score increased from 58.6% in free-text reports to 91.3% in structured-template reports, with better descriptive documentation of mesorectal fascia and circumferential resection margin status, depth of extramural spread, extramural vascular invasion, lateral pelvic nodal assessment, and low rectal sphincter complex involvement. Because no formal statistical hypothesis testing was performed, these findings should be interpreted descriptively rather than inferentially.

The main practical value of the structured template was that it was associated with fewer omissions of clinically relevant positive and negative findings and encouraged a consistent MRI-based risk summary. This is important because rectal MRI reports are used directly in multidisciplinary decision-making, including neoadjuvant treatment planning, surgical approach, sphincter preservation assessment, and anticipated margin risk. Although this audit did not evaluate treatment outcomes or pathological concordance, the descriptive findings suggest that a structured reporting template may be a useful quality-improvement tool for rectal cancer MRI reporting. Wider use of structured templates may improve communication between radiologists, colorectal surgeons, radiation oncologists, and medical oncologists, but further studies are needed before stronger conclusions can be drawn.
